# Dissociative Identity Disorder and the Law: Guilty or Not Guilty?

**DOI:** 10.3389/fpsyg.2022.891941

**Published:** 2022-08-09

**Authors:** Stefane M. Kabene, Nazli Balkir Neftci, Efthymios Papatzikis

**Affiliations:** ^1^Department of Psychology, Canadian University Dubai, Dubai, United Arab Emirates; ^2^Department of Early Childhood Education and Care, Oslo Metropolitan University, Oslo, Norway

**Keywords:** DID, dissociation, legal, responsibility, NGRI-DID

## Abstract

Dissociative identity disorder (DID) is a dissociative disorder that gained a significant rise in the past few decades. There has been less than 50 DID cases recorded between 1922 and 1972, while 20,000 cases are recorded by 1990. Therefore, it becomes of great significant to assess the various concepts related to DID to further understand the disorder. The current review has a goal of understanding whether an individual suffering from DID is legally responsible for the committed crime, and whether or not he or she can be considered competent to stand trial. These two questions are to be raised in understanding DID, by first shedding a light on the nature of the disorder and second by examining the past legal case examples. Despite the very nature of the disorder is characterized by dissociative amnesia and the fact that the host personality may have limited or no contact with the alters, there is no consensus within the legal system whether the DID patients should be responsible for their actions. Further to that, courts generally deny the insanity claims for DID suffering patients. In conclusion, more studies in the field are suggested to incorporate primary data into research, as the extensive reliance on secondary data forces us to believe the conclusions that were previously made, and no opportunity to verify those conclusions is present.

## Introduction

**FIGURE 1:**
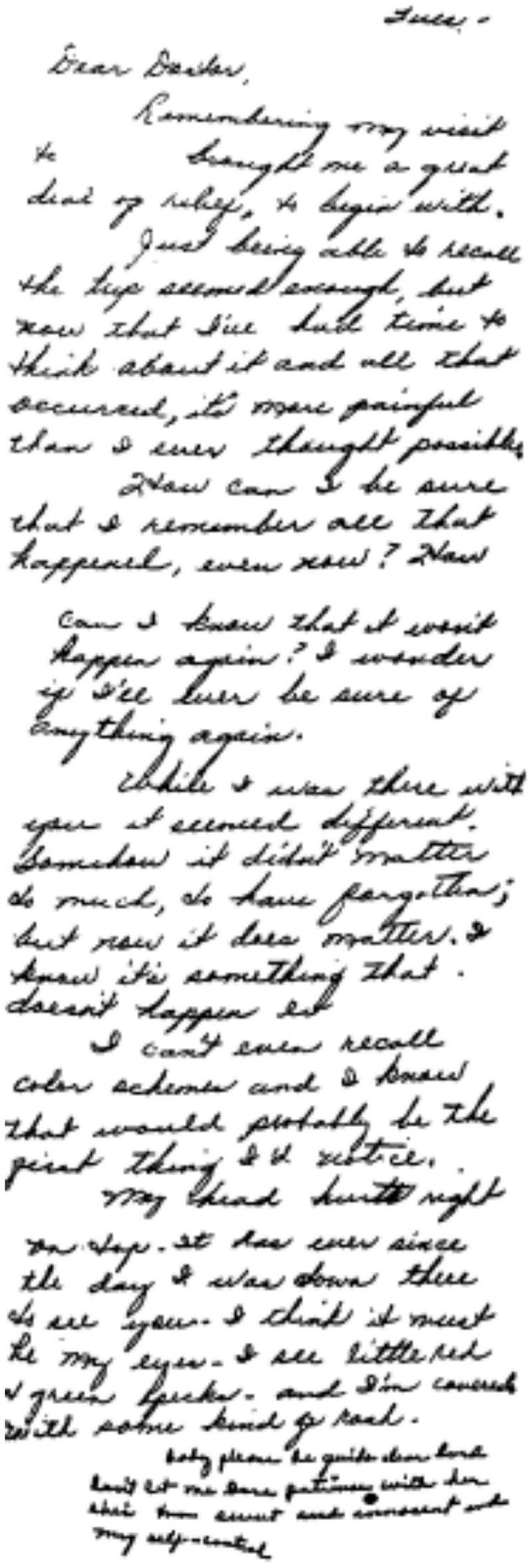
The letter sent to the psychiatrists involved in the case of Chris Sizemore by the patient, Mrs. Sizemore. The sudden change in the handwriting can be observed in the last paragraph of the letter.

Dissociative identity disorder (DID) is classified by DSM-V as “presence of two or more distinct identities or personality states, each with its own patterns of perceiving, thinking, and relating to the environment and the self” where “at least two of these identities or personality states recurrently take control of the person’s behavior” (American Psychiatric Association, 2013). The fact that the DID patients’ multiple identities not only perform differently on personality tests, but also on IQ tests was long since discovered by the predecessor studies in 1950s. It has been also shown that the identities may also differ in age, gender, preferences, and even handwriting (see Figure 1; Thigpen and Cleckley, 1954). Such gigantic difference between the identities and the fact that some identities may not be aware of others’ doings raises the question of legal responsibility of a person suffering from DID should a law be violated by one of the identities within him.

Another legal issue concerned with DID is competence to stand trial. As minor identities may “come out” during the process of the trial, and the dominant identity may have no awareness and/or memory of the actions and events that took place under control of minor identities, it becomes unclear of whether the person under trials is able to completely understand all the happenings during the trials. If that is the case, the question may arise as of person’s competency to stand the trial, not even the legal liabilities that he must incur given the crimes committed. To this aim, the initial attempt will be the examination of the clinical and neuropsychological characteristics of DID by the screening of recent studies that contributed to a better understanding of the disorder. This will provide a foundation for the framework that would in its turn attempt to define whether DID should be in all instances considered a valid reason for the person’s incompetence to stand trial or the person’s legal non-liability for the crime. Subsequently, the current review will examine the existing cases in which the DID patients had to face trials and the sentences they were or were not given. The paper will also attempt to formulate the conditions under which such patients are proven insane, based on the above-mentioned cases. The paper will then analyze the existing materials covering the encounters of DID patients who have committed crimes with the courts and the law enforcement system in general. A further emphasis will be given to the criteria that the courts use when dealing with issue of calling an DID patient to the legal liability. The extent to which the existing laws protecting criminals with mental issues can be applied particularly to DID patients will constitute a part in the current paper. Based on the review performed, the comparison will be made on how well the suggested framework aligns with the current tendencies in law enforcement on sentencing or not sentencing patients suffering from DID.

Current review will only focus on the analysis of secondary data due to the rarity of legal cases concerning DID patients. Therefore, the amount of cases will be too limited to find distinctive patterns in the features of DID symptoms, and the framework suggested will not be as comprehensive, hence, it will not be able to provide reasonable suggestions to the users. Despite using the secondary data as a source of information for the analysis, it must be noticed that the amount of trials involving DID is still very limited. Therefore, another focus of the paper will be on finding the traits in the symptoms of the DID patients not violating the law and hence not standing a trial.

## Dissociative Phenomenology and Dissociative Identity

Dissociative identity disorder, formerly called multiple personality disorder, was first classified in DSM-III-R (American Psychiatric Association, 1987). As it has been seen more commonly in the past 20 years among patients, DID remained as an Axis I disorder in DSM-IV-TR with the renaming of multiple personality disorder to DID (American Psychiatric Association, 2000). Both in DSM-III-R and DSM-IV-TR the diagnostic criteria were laud and clear for that times, but in the context of the current information regarding DID they seem quite sketchy. Unsurprisingly, in 1999, in a survey of board certified psychiatrists in the United States only 21% reported that there is an evidence for DID’s scientific validity (Gharaibeh, 2009). Apparently, the lack of consensus was not an issue in the legal system but also among the mental health care professionals as well. In an effort to overcome this issue, in 2013, American Psychological Association Work Group has proposed slight changes in the diagnostic criteria for DSM-5, in which “the symptoms of disruption of identity may be reported as well as observed, and that gaps in the recall of events may occur for everyday and not just traumatic events” (American Psychiatric Association, 2013). Furthermore, as time progressed, more scientific evidence is being provided by the recent studies scrutinizing the experience of dissociation and its manifestation as DID, leaving little room for any clinical disagreement.

Current definition of dissociation refers to a detachment from one’s sensory experiences, thoughts, feelings, sense of identity or personal history, that occur in reaction to a traumatic experience (Pollock et al., 2017). Dialectical in nature, on one hand it serves as a coping strategy that allows individuals to distance themselves from a trauma that may otherwise be unbearable, on the other it prevents an integration between the trauma and personal narrative, which is a must for the recovery. By manifesting itself in various forms, in depersonalization and derealization an individual has difficulty in processing information about the self and the reality at the present time. In dissociative amnesia the traumatic memory is reserved away from one’s memories and can only be recalled by dissociative flashbacks. In DID, the traumatic information is stored in different parts of the identity, so called alters. Among the others DID manifests the most complex clinical portrait, that is predisposed by prolonged childhood trauma (Ozturk and Sar, 2016). According to Loewenstein and Putnam (1990), the stories of male and female patients had a root from the past where 60% of females had causes related to sexual abuse whereas, 17% of male patients suffered from violence or rape in childhood. In description, DID is characterized by the coexistence of the host and alter identities that are fragmented from each other with limited or no communication. A recent theory identified 13 alter identities, namely, the apparently natural, helper, persecutor, child, gay/lesbian, messenger, abuser (perpetrator), leader (guide, wise), objective (neutral), reversible, talented, suicidal-depressive and potent female, all of which have varying awareness by the host personality. Furthermore, three forms of awareness of the personalities by the host personality were identified: (1) mutual amnesic, (2) unidirectional amnesic, and (3) co-conscious. Although there might be a co-conscious awareness between an alter and the host personality, the degree of recognition between the alter personalities is quite limited. This is due to the fact that each alter contains varying degrees of awareness of the traumatic memories and experiences his or her subjective reality accordingly, resulting in a dissociative barrier (Ozturk and Sar, 2016; Ozturk, 2021).

Several comorbidities are reported among patients with DID, including major depression, somatization disorder and borderline personality disorders, which are among the most commons. Auditory hallucinations, dissociative amnesia, flashbacks and childhood abuse/neglect are other features seen in patients with DID, which are overlapping with the symptoms of other conditions such as PTSD and Schizophrenia. Particularly, Schizophrenia and DID overlap in psychotic symptoms, Schneiderian first rank symptoms in particular, as well as in their traumatic antecedents. However, the differentiation between DID and schizophrenia can be made along several criteria. For instance, poor reality testing and insight of the disorder are observed in schizophrenia, whereas both reality testing and insight remains intact in DID. Unlike schizophrenia, visual hallucinations are quite rare among patients with DID. Schizophrenia is characterized by loose associations with inappropriate affect, however, DID patients manifest conjectural associations with appropriate affect. Still, many patients receive different diagnoses because of lack of awareness of this condition (Tschoke et al., 2011). As for the etiopathology of DID very little is known and the studies in this field are just a few. However, there is recent evidence demonstrating neuroanatomical differences between DID patients and health controls. The research revealed that cortical and subcortical volumes in the hippocampus, amygdala, parietal structures that are responsible for perception and personal awareness as well as frontal structures, which is responsible for movement execution and fear learning were significantly smaller in DID patients. Furthermore, in DID patients larger white matter tracts were detected, which is involved in information communication between somatosensory association areas, basal ganglia, and the precuneus. It was concluded that such neuroanatomical differences might be responsible for some of the symptoms of DID such as host dissociation and neurotic defense mechanisms (Blihar et al., 2020). Apparently, more studies need to be conducted in order to reveal the etiopathology of DID for the recognition of the disorder both legally and clinically.

Despite the complexity of its nature, there are promising treatment models proposed by various professionals, who have a long-standing experience with DID patients (e.g., Kluft, 1999; Pollock et al., 2017). Trauma Based Alliance Model Therapy (TBAMT), for instance, provides a detailed theoretical framework in conceptualizing DID and proposes an evidence-based psychotherapy intervention techniques with a detailed psychotherapy protocol. By proposing an eclectic approach, TBAMT highlights the critical importance of forming therapeutic alliance with the host and all of the other alter personalities. This is for the fusion of each of the alter with the host personality so as to neutralize the traumatic experience by integrating the trauma related autobiographical memories of the alters, which the degree and the content varies for each of them (Ozturk, 2021).

As can be concluded, within the last decade, the scientific advancements in understanding of DID has improved significantly. Integration of the recent clinical findings in the legal system would contribute to a consensus regarding whether claims for NGRI-DID can be accepted. Still, there is an incomprehensible challenge in the forensic assessment of DID patients claiming for the reason of insanity for crimes based on a dissociated state (Farrell, 2011a).

## Dissociative Identity Disorder in the Courtroom

In order to understand the complexities of DID and its relationship with law, one should start the examination by starting from its history (Table 1). The first encounter with DID has taken its place in 1815, when a patient, Mary Reynolds, who, according to Rayna L. Rogers, “might sleep eighteen hours a day and then awaken with large discrepancies in her memory, penmanship and disposition” (Rogers, 1991). The first case of DID that has attracted significant public attention was that of Chris Sizemore, a story of a female patient diagnosed with DID, presented in 1954 by Thigpen and Cleckley. In this case, patient suffering from DID had two very distinctive identities, named by the psychiatrists as Eve White and Eve Black. In Eve’s case, Eve White could be considered as a dominant personality, as Eve Black’s appearances were relatively rare prior to the beginning of the treatment. Eve White and Eve Black had remarkably different behavioral traits. Furthermore, Eve White had no memories of actions done by Eve Black, while Eve Black had a complete awareness of Eve White’s expriences. Thigpen and Cleckley (1954) discussed an event, recalled only by Eve Black and the patient’s relatives, on how Eve committed a prohibited act, specifically she was “wandering through the woods to play with the children of a tenant farmer.” In this particular act, Eve Black only appeared to commit the wrongdoing and let Eve White take on the punishment that followed. The case of Eve becomes an argument that would support protecting DID patients from facing the legal liability for the illegal actions committed. If the person (in the above case Eve White) has no memory of the wrongdoing that cannot be explained by the regular forgetfulness, she may not be considered liable for the crime. However, it must be noticed that while Eve White must be considered innocent, Eve Black could not be exempted from the liability, if we consider two of them as separate identities. Eve Black herself is a sane personality that could appreciate the wrongdoing and the consequences that would follow. During the years ahead, this method was proposed and adopted with some courts that have faced DID patients. These courts have classified the distinct identities of DID patient as separate identities, and therefore sentenced only one or several identities that were in a way or other committing to a crime. Steinberg et al. (1993) examined the results of DID patients facing trials and have demonstrated the reasoning applied by the Supreme Court of Hawaii, that deals with DID suspects in a way that “each identity may or may not be criminally responsible for its acts, each must be examined under the ALI (American Law Institute) Modal Penal Code competency test.”

**TABLE 1:** Court cases where DID was claimed as a basis for NGRI.

Case	Year	Charge	Defense	Court ruling
State vs. Milligan	1978	Rape, murder	NGRI-DID	Evidence of DID. The court found her non-guilty
State vs. Maxwell	1979	Murder	NGRI-DID	Evidence of DID. The court found her non-guilty and the patient sent to a psychiatric hospital
State vs. Grimsley	1982	Driving while intoxicated	NGRI-DID	The court found her guilty as the actions of a person with multiple identity are conscious and voluntary
State vs. Maxwell	1988	Bank robbery	NGRI-DID	The court found her guilty due to the replication of a criminal act
State vs. Moore	1988	Murder	NGRI-DID	The court found her guilty and rejected the diagnosis of DID due to the fact that both of her personalities (host and the alter) knew about the crime and actually took an action
State vs. Huskey	1992	Rape, murder	NGRI-DID	The court found him guilty for rape. The murder trial was declared mistrial in 1999
Commonwealth vs. Orndorff	2000	Murder	NGRI-DID	The court denied a motion for a new trial by a defendant to present the evidence of the DID diagnosed after the conclusion of the guilt phase of the original trial

This method of judging several identities, however, contradicts itself. On one hand, only the personality that has committed a crime will be sentenced to a punishment. However, as all the identities in case of DID share one common physical body including the innocent ones, are subject to the punishment given by the court. Saks (1995) has proposed a theory of general non-responsibility of individuals with DID. Saks’s theory treats identities within an DID patient as separate identities, and therefore claims that courts must not hold DID patients responsible for the crimes unless all the identities existing within a person are involved in a crime, meaning they were either committing a crime or could have interfered and prevent the crime but did not. As per Saks, such theory would correlate with the system of jurisprudence that holds that “ten guilty people should go free rather than one innocent person be jailed” (Sinnott-Armstrong and Behnke, 2000). The research conducted by Farrell (2011a) suggests that courts in general do not accept NGRI-DID (not guilty by reason of insanity due to DID) as a justification for non-responsibility. The reasoning for the rejection of DID as a reason is based on several factors. First one is based on the reason that “scientific evidence failed to meet reliability standards.” Second, abnormal states of consciousness is an insufficient allegation to correspond to a mental disorder that could meet the criteria of M’Naghten Rules (i.e., defendants did not know the nature or quality of their actions or, if they did know, they did not know that what they were doing was wrong) (Farrell, 2011b). In response to this, Nakic and Thomas (2012) reported that the British courts that are more indulgent to the diagnosis of DID have used several approaches to assess criminal responsibilities of DID cases. The alter in-control approach is used to assess the mental state of the alter identity, who was in control when the crime was committed. In the each-alter approach all of the alter identities are assessed for their criminal responsibility. Finally, the host approach examines whether the host personality was unable to evaluate the nature and quality of the conduct committed by the alter. The utilization of the aforementioned approaches will be illustrated in some of the following case examples.

Getting back to the courtrooms, the case of Juanita Maxwell that took place in Florida in 1979 was considered as one of the most unusual at that time. Maxwell was working as a hotel maid and was arrested because of the blood on her shoes and a scratch. Apparently, one of the hotel guests, Inez Kelley, was brutally beaten, bitten, and choked to death. Later on, the murderer was diagnosed with DID where she had six identities. In addition, the identity who committed the crime was called Wanda Weston that was asked to stand trial. People were impressed because Juanita was a soft woman with calm behavior, however, Wanda seemed to be more aggressive and violent (McLeod, 1991). Furthermore, she was even laughing when admitted that she killed a person. As she was a woman suffering from DID, the court found her non-guilty and sent the patient to a psychiatric hospital. In 1988, Maxwella was arrested again for committing two bank robberies and claimed that it happened due to not receiving a proper treatment. By that time, Maxwell had seven identities, but Wanda was still pinned as the culprit of the crimes. Finally, she pleaded “no contest” and was released from prison for time served (Levy et al., 2002).

The case of Thomas Huskey that took a place in Knoxville, brought up a broader question of whether DID is a valid defense for the crime. The man viciously killed four women after forcing them to have sex. In addition, he audiotaped himself with a loud and angry voice during the murder. Lawyers claimed that even though Huskey may have been speaking, the words were coming from an alter ego that took control of his actions (Haliman, 2015). Moreover, the defense attorneys claimed that the tape of other personality so-called Kyle is not a proof that Thomas – a soft-spoken and calm man – committed any crime. Prosecutors asked an expert, Dr. Herbert Spiegel, to evaluate the presence of multiple identities and how each could impact the actions made by one human. Interestingly, the vocabulary, tone, and manner of talking were completely different in both identities when the professionals agreed it was the voice of the same person. One of the psychiatrists claimed Huskey was just a good actor and had an incredible ability to manipulate people (Appalachian Unsolved, 2017). The court had only two options: whether find him guilty of the crime or non-guilty due to DID and signs of insanity. No matter how attorneys tried to defend Huskey, the majority of jurors came to a conclusion that he needs punishment for his crimes, and he is currently serving a 64-year sentence.

Speaking of “alter approach” (the approach under which the courts decide on person’ responsibility based on sanity or insanity of the alter in control during the crime), many courts have judged based on these criteria. In case of Grimsley, a woman accused of drunk driving and pleading for NGRI-DID, the court have concluded that “there was only one person driving the car and only one person accused of drunken driving. It is immaterial whether she was in one state of consciousness or another, so long as in the personality then controlling her behavior, she was conscious, and her actions were a product of her own volition. The evidence failed to indicate that Jennifer was unconscious or otherwise acting involuntarily” (Sinnott-Armstrong and Behnke, 2000).

A possible reason that can explain the courts’ tendency to reject the NGRI-DID is the social response to the successful defenses based on that reason. The case in 1978, at which the defendant, Billy Milligan, who was a serial rapist, was found innocent for the reason of insanity (NGRI-DID), found an extreme outrage in the society. Since then, it was a very rare phenomenon to see courts accepting DID as a justification for insanity. Undeniably, the social response to DID hinders the objective judgment of DID-diagnosed patients for their legal responsibility. Certain psychiatrists do not believe in the DID at all, and there is a great suspicion over the ease of malingering DID in order to plead for insanity. The reason behind the thinking is the extreme complexity of symptoms that leads to the difficulty in the scientific evaluation of the patient’s disease.

The research conducted by Nakic and Thomas (2012) presents the case of Goering Orndorff, a woman who has killed her husband and altered a crime scene in a way that the scene presented her actions as a self-defense. During the process of the trials, specialists were asked to evaluate her competency to stand the trial due to the existence of dissociative symptoms. Some of the experts have agreed on DID diagnosis being applied to Mrs. Orndorff and presented their opinions during the trial. However, it was later revealed that the crime scene was intentionally altered, and that Mrs. Orndorff has told her cellmate that she attempts to malinger the DID in order to plead for insanity defense. With the account of all these facts, the court has found her guilty and sentenced her to 32 years of imprisonment. The later motions for new hearing proposed by the defense, were rejected by the courts.

Even though the people diagnosed with DID seem as no danger to the society at first, the statistics conducted by clinicians shows that nearly half of the patients had violent behavior (Webermann and Brand, 2017). Since there is a sign of aggressiveness, the probability of committing a crime is relatively high and hard to be prevented due to a dissimilar behavior under each of the identities. At the same time, psychiatrists claimed that criminals tend to malinger DID in order to be defended by the law of insanity (Saks, 1995). However, faking DID is considerably difficult because the person should be able to completely separate characters and fully control the actions and mind over a prolonged time. According to the case of Ms. Moore, there were two identities that acquiesced in the crime and found responsible for their actions. First of all, Billy Joel was a personality that actually terrorized a group of children and even ended up beating one of them to death. Then, there was the other identity so-called Marie Moore that would actually call herself pretending it is Billy with children’s daily instructions. Moreover, she even deflected the police when under suspicion. In this case, Ms. Moore could not be diagnosed with DID because both of her identities knew about the crime and actually took an action. Apparently, she was not mentally stable and could still have some mitigation but her claim of suffering from DID was completely rejected (Moore, 1988).

Nevertheless, people diagnosed with DID can put not only themselves in trouble but also confuse the others around them by an abnormal change of mood and behavior. The case of Mark Peterson took place in Oshkosh in 1990, however, the psychiatrists found a progressive disorder where the number of identities was increasing and even represented changes of age in the majority of them (Possley, 2014). Mark Peterson was a victim of dealing with a woman diagnosed with DID where she agreed to have intercourse with a 29-year-old man. The identity that emerged during that time was 20 years old when the other 6-year-old identity was watching from a different perspective. Later on, Mark was charged and convicted of second-degree sexual assault because it is illegal to have an intercourse with someone who is mentally ill. In addition, at the time of the incident in June woman had 21 identities, when later during the trial in November prosecutors discovered that this number has increased to 46. Even though Peterson was never retried for the crime after the overturned a month later verdict, the case brought up questions about how to deal with DID victims that claim to be assaulted during the presence of one of the identities.

Another cause of concern, as in the case of Peterson, is taking into consideration how to deal not only with DID patients who committed a crime but also how to punish the people who were interacting illegally and harmed one of the identities (Possley, 2014). The action can be done by one identity and it will be considered acceptable when the other identity will look at that as a crime. However, the same human might not remember doing any of these since the switch of the identities happens naturally and the memory of past actions usually do not interfere with one another. Meanwhile, the prosecutors tend to end the trial faster in order not to put the victim in the position of psychological trauma all over again.

## Discussion and Analysis

The literature review suggests a general tendency from the courts’ side not to accept the DID propositions and hence exempt the person from the responsibility on the basis of NGRI-DID. The major reasons for the tendency were lack of reliability of scientific methods in diagnosing DID, the possibility of a suspect to malinger DID in such a way that certain specialists will give the desired diagnosis (Ms. Orndorff’s case), the social response to the successful defense based on NGRI-DID, and the immaterial fact of DID, as related to the legal responsibility (the alter in control being sane and competent to stand the trial). Moreover, the case of Maxwell clearly showed that the person can commit the crime again when the society will hardly accept the decision of non-guiltiness. Therefore, the prosecutors tend to find criminals responsible due to the past experience and research done on DID.

The complexity of DID is also supported through the differences in the opinions on the reliability of the tests administered with the purpose of diagnosing DID. It has been suggested by Steinberg that the introduction of Structured Clinical Interview for DSM-III-R (SCID) and the Schedule for Schizophrenia for Affective Disorders and Schizophrenia (SADS) has increased the reliability in diagnosing disorders such as DID (Steinberg et al., 1993). The case of Ms. Orndorff, however, has happened in 2000 and suggests that the diagnostic capabilities in terms of DID were still lacking and hence insufficient to accurately diagnose DID.

As was mentioned before, the courts do have a tendency to deny the NGRI-DID claims for the DID patients that commit crimes. However, it becomes interesting to check on whether similar illnesses, such as epileptic seizures, face the same level of denials in the courts. Epileptic seizures resemble DID in terms of legal responsibility in a way that during a seizure, a person may engage in “actions such as picking at the clothes, trying to remove them, walking about aimlessly, picking up things, or mumbling” (Farrell, 2011b). Of greater importance is the fact that “following the seizure, there will be no memory of it” (Farrell, 2011a). As the actions performed during a seizure are involuntary, the person is unable to appreciate the actions or the consequences that follow, and has no memory of the events, not explained by the regular forgetfulness, the court should consider the person insane at the moment of committing a crime. Farrell elaborates on three cases of successful defenses on the basis of “non-insane automatism” (the definition under which courts nowadays classify epileptic seizures). In all cases, the courts have declared the defendants not guilty of the crimes, as their actions were involuntary, and the defendants had no memory of the events.

It is interesting in the light of above-mentioned cases to see the drastic difference in the courts’ opinions about the similar illnesses in terms of legal responsibility. In both cases, the defendants have no memory of the actions committed. However, it must also be presented that DID patients generally have an identity within them that was aware of the wrongdoing and also carries the memory of that wrongdoing, while under epileptic seizures there is not a single trace that would suggest that the defendant has a memory of a wrongful conduct. One could also argue that while considering the epilepsy-suffering patient, we are concerned with a single identity that is a subject to a biological illness and therefore, it becomes easy to say that the person’s actions were indeed involuntary, while considering the DID, we are talking about totally different identities with their own mindset within a single individual with a very limited information regarding its etiopathology. It means that the court can be reasonably confident in the reliability of epilepsy truly belonging to an individual, while an DID patient can potentially malinger the illness. Even though a few studies have emerged within the last a few years investigating the neurological correlates of DID, the research in this domain is still in the stage of infancy.

Taking a look at the root causes of the DID, it is found that severe psychological trauma or prolonged abuse in the childhood are the most possible reasons that cause the brain to trigger the self-defense mechanisms and protect itself through the dissociation of identities. As the effect of DID is not happening on its own and is occurring following a severe trauma, it should be considered a mental illness and thus be a sufficient reason for claiming the person to be not guilty by the reason of insanity (NGRI-DID). Moreover, both genders can be exposed to any kind of assault or negative experience in the childhood and the tendency of being diagnosed with DID of those victims is correlated. Both men and women showed similar types of identities and behavior that leads to the conclusion that crimes can be done by anybody regardless of their sex (O’Boyle, 1993). Therefore, the framework of how to justify or punish the person who committed wrongdoings should be the same for both male and female.

Many psychiatrists tend to question whether the person is really suffering from DID or trying to pretend in order to have NGRI-DID. However, involving only one specialist might not be enough as we all are human beings and think subjectively based on our past experience and beliefs. The case of Thomas Huskey was advised by the psychiatrist that already had strong beliefs that the murderer is just a great actor, therefore, he did not attempt to search for the root cause of the behavior that was hard to explain at that time (Haliman, 2015). Moreover, involving a few professionals is no longer enough since the opinion can differ based on individual observation, however, even the final judgment can be affected by groupthink. Based on the case of Ms. Moore, it was easier to find her guilty since both identities were directly involved in the action, so even the presence of other minor identities would not justify her wrongdoings. In particular, she was not even diagnosed with DID during the trial and was found responsible regardless of her mental illness (Moore, 1988).

Regarding the doubts over the reliability of measures for the assessment of DID, there are so far very few mechanisms available to psychiatrists that can be used in an attempt to evaluate patient’s dissociative disorders. It has been found that the long interviews used during the evaluation allow for emerging of different identities present within an individual. The long aspect of the interviews and evaluation also reduce the possibility of patient malingering the diagnosis. Kluft (1999) stated that “simulated DID presents crude manifestations of the disorder, such as stereotypical good/bad identity states and a preoccupation with the circumstances individual hopes to avoid by obtaining an DID diagnosis.” Kluft also suggested that it is difficult for the individual to maintain the voice, set of body gestures, and memory for every personality that he or she is trying to simulate. Hence, it can be suggested that the actual possibility of malingering DID is extremely challenging, and that cases of malingered DID will be very rare compared to correctly diagnosed DID.

Speaking of suggesting the framework for deciding on person’s liability on the basis of DID, the diagnosis has proven itself to be so complex that no universal method can actually be applied. However, there is a set of actions that should be done in order to assess the responsibility for the crime committed. Initially, an evaluation of the patient should be performed by several independent psychiatrists. The DID in our opinion should only be considered valid when all the psychiatrists involved agree on the opinion that the defendant is suffering from DID. Based on the diagnosis, the question of competency to stand trial must be answered. Then, the court should select the appropriate method for assessing the responsibility. The “host-alter” method is best when there is a dominant personality present, and the crime was committed by the alter identity. The “alter-in-control” method should be used when there is no clear evidence of the dominant identity. If the method used provides a result that supports the fact that the identity evaluated is insane at the time of committing a crime, the defendant should be considered not guilty.

## Limitations

The paper does carry certain limitations. The main limitation lies in the fact that no primary sources of data were used. The nature of literature review exempts researchers from direct interaction with the patients. This is even true for the previous research that our paper is based on. The existing literature primary deals with evaluating the cases that have already happened, and not evaluating the currently open cases. It brings us to the need to believe the judgments of previous psychiatrists involved in the cases, not being able to actually see the patients and whether or not the researchers would agree on the diagnosis and the responsibility with the psychiatrists involved. The suggestion for future research that arises from this limitation is to attempt to conduct the study that would be based on the primary data by conducting interviews with specialists and patients involved or conducting observations. Case study method could be suggested.

Secondly, the paper primary deals with the cases from the Western region. It raises the question of the ability to generalize the results to the other region, as different cultures have different approaches toward legal judgments. It would hence be interesting to see the results of similar studies in the Asian and Eastern regions to compare whether these regions possess the similar views on the topic of multiple personality disorder. The future research on the above-mentioned areas of the world will therefore be of importance and value to the field of literature currently available.

## Recommendations

The research has identified few critical areas in the field of DID that have not yet been addressed by the previous research and are also not addressed by our research. Previous research has either involved case studies of non-criminal DID patients, or analysis of criminal DID patients that was done after the trials have been concluded. However, it is of great importance to conduct the study that would examine the criminal DID patients while trials and evaluations are still ongoing. Such study would tackle the limitations present in our paper, as well as ones from previous research.

Future researchers are also encouraged to compare the courts’ views on DID with other disorders, similarly to our paper’s comparison of DID to epileptic seizures. Such studies are of interest to the field of psychology, as they may change the opinions on the diagnosis from the law enforcement agencies, if they see that similar disorders are treated differently, just like DID and epileptic seizures. Moreover, the research paper was focused on the cases that happened in the West and under its legal environment. The further research is suggested to look at the wider aspect of countries and nationalities, however, the availability of secondary source data as of now is really limited.

## Conclusion

Dissociative identity disorder is a complex and controversial disorder which has seen opposing opinions on the existence of the disorder itself and concepts associated with it, such as the legal responsibility of the defendants suffering (or appearing to suffer) from multiple personality disorder.

The paper has examined the existing literature on the topic of multiple personality disorder and has found a general courts’ tendency to not accept DID as a reason to justify the defendant’s insanity and hence not to exempt the person from the legal responsibility. In part, such tendency is explained by the negative social reaction to the cases where defendants were found not guilty by the reason of insanity (see Milligan’s case). Another explanation for the tendency is the controversial and subjective nature of DID and differences in the opinions held by psychiatrists when evaluating a person on whether DID diagnosis could be given.

Based on the existing literature, the paper has suggested the basis for the framework on which the legal systems can standardize their approach toward DID. It has to be noted, however, that the framework still cannot be made universal, because the symptoms and traits existing differ from one patient to another (for example, the existence of the dominant personality). The induction of hypnosis during the course of treatment makes the issue even more complex, as we have seen from the case of Eve, where Eve White was a dominant personality until hypnosis sessions began and Eve Black learned to emerge at her will.

Based on the found secondary source data, the progress of developing the legal framework has improved when the awareness of DID keeps increasing, respectively. The courts tend to find DID criminals responsible for their actions due to the social factor and previous evidence. The approach of judgment is not related to the gender of the person since both male and female share the same types of identities. Even though the evaluation of DID is done by the psychological measures, the questions whether some people actually fake this disease keep appearing. Therefore, the involvement of the latest methods and a group of psychiatrists during the trial showed a positive effect on the final judgment.

## Author Contributions

SK: conceptualization and supervision. SK and NB: data curation and writing – original draft. SK, NB, and EP: methodology and writing – review and editing. All authors have read and agreed to the published version of the manuscript.

## Conflict of Interest

The authors declare that the research was conducted in the absence of any commercial or financial relationships that could be construed as a potential conflict of interest.

## Publisher’s Note

All claims expressed in this article are solely those of the authors and do not necessarily represent those of their affiliated organizations, or those of the publisher, the editors and the reviewers. Any product that may be evaluated in this article, or claim that may be made by its manufacturer, is not guaranteed or endorsed by the publisher.

## References

[B1] American Psychiatric Association (1987). *Diagnostic and Statistical Manual of Mental Disorders (3th ed., text rev).* Washington, DC: American Psychiatric Association.

[B2] American Psychiatric Association (2000). *Diagnostic and Statistical Manual of Mental Disorders (4th ed., text rev).* Washington, DC: American Psychiatric Association.

[B3] American Psychiatric Association (2013). *Diagnostic and Statistical Manual of Mental Disorders (5th ed).* Arlington, VA: American Psychiatric Association. 10.1176/appi.books.9780890425596

[B4] Appalachian Unsolved (2017). *The Serial Killer who got away with Murder.* Available Online at: https://www.wbir.com/article/news/crime/appalachian-unsolved-the-serial-killer-who-got-away-with-murder/51-409311086.

[B5] Blihar D. Delgado E. Buryak M. Gonzalez M. (2020). A systematic review of the neuroanatomy of dissociative identity disorder. *Eur. J. Trauma Dissociation* 4 100–138. 10.1016/j.ejtd.2020.100148 33433297

[B6] Farrell H. (2011a). Dissociative identity disorder: medicolegal challenges. *J. Am. Acad. Psychiatry Law* 39 402–406. 21908758

[B7] Farrell H. (2011b). Dissociative Identity Disorder: no excuse for criminal activity. *Curr. Psychiatry* 10 33–40.

[B8] Gharaibeh N. (2009). Dissociative identity disorder: time to remove it from DSM V? *Curr. Psychiatry* 8 30–36.

[B9] Haliman A. (2015). *Using Multiple Personality Disorder as Legal Defense.* Available Online at: https://abcnews.go.com/Primetime/story?id=132119&page=1.

[B10] Kluft R. (1999). An overview of the psychotherapy of the dissociative identity disorder. *Am. J. Psychol.* 53:3, 289–319. 10.1176/appi.psychotherapy.1999.53.3.289 10586296

[B11] Levy A. Nachshon D. Carmi A. (2002). *Psychiatry and Law*, 1st Edn. Yozmot Heiliger Ltd.

[B12] Loewenstein R. Putnam F. (1990). The clinical phenomenology of males with DID: a report of 21 cases. *Dissociation* 3 135–143.

[B13] McLeod M. (1991). *Wanda: the part of me that is a Murderer.* Available Online at: http://www.astraeasweb.net/plural/juanita3.html.

[B14] Moore S. V. (1988). *The Supreme Court of New Jersey.* Available Online at: https://law.justia.com/cases/new-jersey/supreme-court/1988/113-n-j-239-1.html.

[B15] Nakic M. Thomas P. (2012). Dissociative Identity Disorder in the Courtroom. *J. Am. Acad. Psychiatry Law* 40 146–148.

[B16] O’Boyle M. (1993). Personality Disorder and Multiple Substance Dependence. *J. Pers. Disord.* 7 342–347. 10.1521/pedi.1993.7.4.342

[B17] Ozturk E. (2021). Trauma based alliance model therapy. *Med. Sci.* 10 631–650. 10.5455/medscience.2021.03.100

[B18] Ozturk E. Sar V. (2016). Formation and Functions of Alter Personalities in Dissociative Identity Disorder: A Theoretical and Clinical Elaboration. *J. Psychol. Clin. Psychiatry* 6:385. 10.15406/jpcpy.2016.06.00385

[B19] Pollock B. E. Macfie J. Elledge C. (2017). Evidence for phase based psychotherapy as a treatment for dissociative identitiy disorder comorbid with major depressive disorder and alcohol dependance. *J. Trauma Dissociation* 18 595–609. 10.1080/15299732.2016.1241853 27689689

[B20] Possley M. (2014). *Mark Peterson - National Registry of Exonerations.* Available Online at: https://www.law.umich.edu/special/exoneration/Pages/casedetail.aspx?caseid=4512.

[B21] Rogers R. L. (1991). Multiple Personality and Chanelling. *Jefferson J. Psychiatry* 9 3–13. 10.29046/JJP.009.1.001

[B22] Saks E. (1995). The criminal responsibility of people with multiple personality disorder. *Psychiatr Q.* 66 119–131. 10.1007/BF02238859 7652095

[B23] Sinnott-Armstrong W. Behnke S. (2000). Criminal Law and Multiple Personality Disorder: The Vexing Problems of Personhood and Responsibility. *South. Calif. Interdiscip. Law J.* 14 277–296.

[B24] Steinberg M. Bancroft J. Buchanan J. (1993). Multiple Persinality Disorder in Criminal Law. *J. Am. Acad. Psychiatry Law* 21 345–356. 8148515

[B25] Thigpen C. H. Cleckley H. (1954). A case of multiple personality. *J. Abnorm. Soc. Psychol.* 49 135–151. 10.1037/h0057795 13128975

[B26] Tschoke S. Uhlmann H. Steinert T. (2011). Schizophrenia or trauma-related psychosis? Schneiderian first rank symptoms as a challenge for differential diagnosis. *Neuropsychiatry* 1 349–360. 10.2217/npy.11.27

[B27] Webermann A. Brand B. (2017). Mental illness and violent behavior: the role of dissociation. *Borderline Pers. Disord. Emot. Dysregulation* 4:2. 10.1186/s40479-017-0053-9 PMC5260135 28138388

